# Effects of partial or full replacement of soybean meal with urea or coated urea on intake, performance, and plasma urea concentrations in lactating dairy cows

**DOI:** 10.1111/jpn.14034

**Published:** 2024-08-13

**Authors:** Rainer Rauch, Kelly Nichols, Isabela P. C. de Carvalho, Jean‐Baptiste Daniel, Javier Martín‐Tereso, Jan Dijkstra

**Affiliations:** ^1^ Trouw Nutrition R&D Amersfoort The Netherlands; ^2^ Animal Nutrition Group Wageningen University and Research Wageningen The Netherlands

**Keywords:** ammonia toxicity, human‐edible protein efficiency, nitrogen efficiency, non‐protein nitrogen, urea toxicity

## Abstract

We expected mitigation of the hypophagic effects of urea (U) with a coated urea (CU) product that aimed to partially shift urea supply to the post‐ruminal gastrointestinal tract. Ruminal release and post‐ruminal digestibility of CU was evaluated in vitro, followed by a randomised complete block experiment (54 Holstein‐Friesian cows; 177 ± 72 days in milk). Soybean meal (SBM) was partially (PR) or fully (FR) replaced on an isonitrogenous basis by beet pulp and U or CU. Urea sources were included at 12 (U‐PR, CU‐PR) and 19 (U‐FR, CU‐FR) g/kg dietary dry matter (DM). Hypophagic effects were similar for U‐PR and CU‐PR (−11% vs. −7%), and for U‐FR and CU‐FR (−13% vs. −12%) compared with SBM (average 25.8 kg DM intake/d). Compared with SBM, U‐PR and CU‐PR reduced yields of milk (−8%) and protein (−12%), U‐PR reduced yield of fat (−9%) and fat‐ and protein‐corrected‐milk (FPCM; −9%), and CU‐PR tended to reduce FPCM yield (−5%). Compared with SBM, U‐FR and CU‐FR respectively reduced yields of milk (−21%, −22%), protein (−25%, −26%), fat (both −14%), lactose (−20%, −21%), and FPCM (−17%, −19%), and lowered N (−15%, −12%) and feed (−8%, trend, −9%) efficiency. Human‐edible protein efficiency approximately doubled with U‐PR and CU‐PR and approximately tripled with U‐FR and CU‐FR compared with SBM. Milk composition and plasma urea concentration were similar between U and CU, except for a trend for a greater plasma urea concentration with U‐PR compared with CU‐PR. Dry matter intake patterns differed for CU‐PR compared with U‐PR and for CU‐FR compared with U‐FR, suggesting effects of urea release rate or location on feeding behaviour. Overall, replacing SBM with U or CU reduced DM intake and milk production and affected nutrient efficiencies. Coated urea influenced DM intake pattern but did not affect total DM intake or milk production compared with U.

## 1 INTRODUCTION

Ruminants have the capacity to use non‐protein nitrogen (NPN) for microbial protein synthesis, and in an extreme example, NPN was the sole dietary nitrogen (N) source for lactating dairy cows (Virtanen, 1966). Endogenous urea recycling plays an important role in the N economy of ruminants (Lapierre & Lobley, 2001; Nichols et al., 2022), and is positively associated with reduced ruminal ammonia levels (Abdoun et al., 2006; Li et al., 2019) and increased plasma urea levels (Sunny et al., 2007; Vercoe, 1969; Weston & Hogan, 1967). Endogenous urea can contribute to metabolizable protein supply if it is recycled to the rumen and stimulates microbial protein synthesis. Average hepatic urea production can account for ~72% of N intake (Batista et al., 2017), and on average ~67% of urea synthesised in the liver will be recycled to the gut (Lapierre & Lobley, 2001). However, recommendations are to feed no more than 20% of total dietary N as NPN (Kertz, 2010). Limitations to dietary urea inclusion level include a lower efficiency of ruminal microbial protein synthesis with ammonia compared with amino acid sources (Dijkstra et al., 1998), potential ammonia toxicity, and hypophagic effects at high dietary urea inclusion levels (Brito & Broderick, 2007; Kertz, 2010; Poos et al., 1979). Maintaining ruminal ammonia at relatively low and stable levels may offer an opportunity to reduce hypophagic effects of urea and increase dietary urea inclusion levels. Notably, urea derivatives that resist ruminal degradation (e.g., biuret, isobutylidene diurea) have been shown to reduce post‐prandial peaks of ruminal ammonia relative to urea (Komatsu & Sakaki, 1971; Smith, 1986; Veen & Bakker, 1977).

Several studies have evaluated the potential utility of supplementing urea post‐ruminally. In sheep, post‐ruminal urea infusion compared with the negative control (infusion of sodium phosphate solution), or partial replacement of dietary urea with post‐ruminal urea infusion, increased digestibility (Egan, 1965) or intake (Becker et al., 1982; Egan, 1965). More recently, Oliveira et al. (2020) reported that DMI did not differ between continuous infusions of urea into the rumen or abomasum of non‐lactating heifers, but the proportion of microbial N originating from recycled urea increased with abomasal urea compared with ruminal urea. Nichols et al. (2023) investigated increasing doses of urea via post‐ruminal infusions in lactating dairy cows and observed a quadratic response in dry matter intake (DMI), where DMI increased 11% compared to the negative control (water infusion) when the dose of infused urea was equivalent to 0.7% of DMI. In a study with non‐lactating heifers, rumen ammonia concentrations were lower, rumen pH was more stable, and apparent total tract neutral detergent fibre (NDF) digestibility increased in response to continuous abomasal urea infusion compared to a ruminal pulse‐dose of urea (de Carvalho et al., 2020). Together, these studies indicate that post‐ruminal urea may supply N to the rumen for microbial protein synthesis, that intake may be influenced by location or rate of urea supply, or both. Adjacent to studies using post‐ruminal infusion models, several studies with lactating and growing cattle have investigated the effect of urea products that have been coated to slow their degradation in the rumen (i.e., slow‐release urea) on DMI, digestibility, and N metabolism in comparison to traditional urea (Highstreet et al., 2010; Taylor‐Edwards et al., 2009; Xin et al., 2010) or plant proteins (Miranda et al., 2019; Sinclair et al., 2012). In contrast to post‐ruminal infusion, slow‐release urea products are designed to still release the entirety of urea in the rumen.

Based on the limitations of dietary urea feeding and the potential utility of post‐ruminal urea supplementation, we hypothesised that hypophagic effects of urea would be mitigated by a coated urea (CU) product designed to partially shift urea supply to the post‐ruminal gut. Our objective was to characterise the effects of replacement of soybean meal (SBM) with urea or CU on DMI, milk production, nutrient efficiency, and plasma urea concentrations in dairy cows.

## 2 MATERIALS AND METHODS

This study was conducted at the Dairy Research Facility of Trouw Nutrition Research and Development (Kempenshof, Boxmeer, the Netherlands). All experimental procedures were approved by the Central Committee of Animal Experiments (the Hague, the Netherlands) and conducted under the Dutch Act on Animal Experiments, which complies with European Directive 2010/63/EU.

### 2.1 Animals, experimental design, and treatments

**Figure 1:**
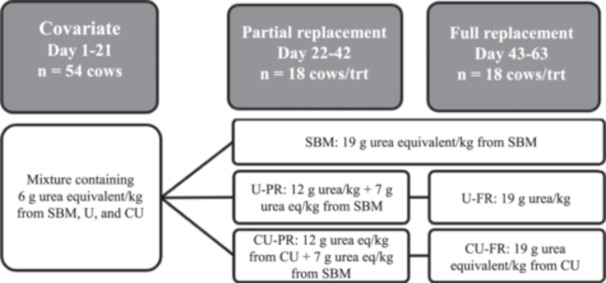
Design of the experiment evaluating the impact of iso‐nitrogenous partial or complete replacement of soybean meal (SBM) with urea (U) or coated urea (CU). The diet fed during the 21‐day covariate period contained an equal contribution (6 g urea equivalent/kg) from SBM, U, and CU. Cows were blocked according to parity, days in milk, and dry matter intake at the end of the covariate period. Partial replacement of SBM with U (U‐PR) or CU (CU‐PR) was at 12 g urea equivalent/kg and full replacement of SBM with U (U‐FR) or CU (CU‐FR) was at 19 g urea equivalent/kg (all dry matter basis).

Fifty‐four Holstein‐Friesian dairy cows (177 ± 72 days in milk at the start of the experiment; 9 primiparous and 45 multiparous, 2.8 ± 1.3 lactations) were used in a randomised complete block design (Figure 1). The first 21 days served as a covariate period, where cows were offered a TMR containing 6 g urea equivalent/kg (DM basis) of SBM, U and CU, respectively. Milk composition data collected during morning and evening milking of days 19 and 21, respectively, were used to calculate milk covariates per cow. Similarly, feed intake from days 15 to 21, obtained with 30 roughage intake control (RIC) feed bins (Insentec, Marknesse, the Netherlands), was used to compute a DMI covariate per cow. Cows were blocked according to parity, days in milk, and DMI (18 blocks) and assigned randomly within a block to one of three treatments. From day 22 to 42 (partial replacement (PR) period), cows received their assigned TMR formulated with either SBM, U (U‐PR), or CU (CU‐PR). Urea or CU were included at 12 g urea equivalent/kg dietary DM during the PR period. From day 43 to 63 (full replacement (FR) period), cows on SBM remained on the same formulation whereas cows receiving U‐PR and CU‐PR were switched to a formulation where U (U‐FR) and CU (CU‐FR) were included at 19 g urea equivalent/kg on a DM basis. The difference in N contribution between the urea source and SBM was compensated by the addition of beet pulp such that the U and CU treatments were isonitrogenous with SBM at both PR and FR. The coating of CU was compensated in U and SBM by the addition of rumen‐protected fatty acids. The composition of the treatment diets is presented in Table 1. The CU diets were formulated to be relatively deficient in rumen degradable protein compared with rumen fermentable energy, as evidenced by the slightly negative average rumen protein balance (OEB levels ‐6 and ‐13 g/kg DM for CU‐PR and CU‐FR, respectively; Table 1). This was intended to stimulate urea recycling, as urea flux into the rumen is positively associated with fermentable energy and negatively related to rumen ammonia concentration (Abdoun et al., 2006; Kennedy & Milligan, 1980).

**Table 1:** Ingredient and chemical composition of diets^a^

	Concentration (g/kg of DM, unless otherwise stated)					
	Partial replacement			Full replacement		
Composition	SBM	U‐PR	CU‐PR	SBM	U‐FR	CU‐FR
Ingredient						
Maize silage	492	491	492	476	478	478
Grass silage	96	96	97	97	97	97
Soybean meal^b^	129	43	43	134	—	—
Beet pulp	123	196	195	128	239	239
Wheat	122	123	123	127	127	127
Urea	—	12	—	—	19	—
Coated urea	—	—	14	—	—	21
Limestone	8.3	6.5	6.5	8.6	5.9	5.9
Sodium bicarbonate	7.4	7.4	7.4	7.6	7.7	7.7
Molasses (beet)	6.1	6.2	6.1	6.3	6.4	6.4
Monocalcium phosphate	3.3	5.6	5.5	3.4	6.9	6.9
Magnesium sulphate, anhydrous	3.0	3.6	3.6	3.1	4.1	4.1
Rumen‐protected fat^c^	2.3	2.3	0.8	2.4	2.4	—
Salt	2.6	2.4	2.4	2.7	2.3	2.3
Vitamin/mineral premix	2.0	2.0	2.0	2.1	2.1	2.1
Magnesium oxide	1.5	1.5	1.5	1.5	1.5	1.5
Live yeast^d^	0.02	0.02	0.02	0.02	0.02	0.02
Nutrient composition						
DM, g/kg as fed	473	474	473	458	458	458
OM	915	915	914	916	915	912
CP	160	161	158	161	163	156
CP, excluding urea	160	131	126	161	115	108
Crude fat	25	25	25	27	25	25
NDF	300	318	324	298	340	331
ADF	167	175	176	165	183	183
ADL	9	10	10	8	10	9
Starch	218	221	228	220	223	226
Total sugars	27	28	21	32	24	26
NE_L_, MJ/kg DM^e^	6.8	6.6	6.6	6.8	6.5	6.6
DVE^f^	101	84	84	102	77	77
OEB^g^	10	21	‐6	11	27	−13

^a^ Soybean meal (SBM) was partially replaced with urea and beet pulp (U‐PR) or with coated urea and beet pulp (CU‐PR) during the partial replacement period, or fully replaced with urea and beet pulp (U‐FR) or with coated urea and beet pulp (CU‐FR) during the full replacement period.

^b^ Solvent‐extracted soybean meal containing 480 g CP/kg product.

^c^ Megalac, Volac International, Hertfordshire, UK.

^d^ Levucell SC 20, containing 2 × 10^10^ colony forming units/g, Lallemand, Blagnac, France.

^e^ Calculated with the Dutch NE system (CVB Centraal Veevoederbureau, 2018) based on TMR ingredient composition.

^f^ Intestinal digestible protein. Calculated with the Dutch DVE/OEB system (CVB Centraal Veevoederbureau, 2018) based on TMR ingredient composition.

^g^ Rumen degradable protein balance. Calculated with the Dutch DVE/OEB system (CVB Centraal Veevoederbureau, 2018) based on TMR ingredient composition, and in the case of CU, the in vitro estimate of ruminal release (Table 2).

Treatments were delivered through RIC bins beginning on day 22, where all cows within a specific treatment had access to all RIC bins (10 bins per treatment) allocated to that respective treatment diet. Cows were assigned to their respective RIC feed bins on day 21. Cows were housed in a free stall barn with free access to fresh water and electronic access to their RIC bins. Cows were fed daily at 0900 h throughout the entire study, and remaining feed was removed from the bin each day before fresh feed allocation. The start and end weight of the feeder was recorded at every visit of a cow to a feeder. The RIC bins were checked daily for correct functioning.

### 2.2 In vitro product evaluation

The CU product was a non‐commercial prototype (Trouw Nutrition R&D, Amersfoort, the Netherlands) manufactured by coating urea (Yara, Brunsbüttel, Germany) with hydrogenated fat layers (hydrogenated palm oil; predominantly C16:0 and C18:0), aiming to extensively reduce rumen solubilisation of urea, while maintaining total tract digestibility (European Patent Office International Application No. PCT/EP2018/076234; Häussner et al., 2020). To quantitatively describe the nutritional qualities of this prototype before the animal experiment, the CU product was subjected to an in vitro evaluation to estimate ruminal protection and post‐ruminal digestibility (Häussner et al., 2020). Since the solubility of urea is virtually complete in rumen fluid and hydrogenated fat is practically insoluble, weight loss is a simple method for determining the rumen protection of CU. To simulate rumen stability, 2.5 g of pelletized ruminant feed (without urea and containing 10% CP on DM basis) was placed into 1000 ml Schott flasks containing 250 ml of McDougall's buffer solution at pH 6.0. Ankom nylon bags were filled with 5 g of CU and placed in the flasks along with the pelleted feed and buffer solution. The flasks were incubated for 6 h (as an estimate of liquid retention time in rumen) at 39°C at 100 rpm with an amplitude of 25 mm (horizontal circular motion). After incubation, the nylon bags were removed from the flasks, washed with cold water and dried with air exchange at 39°C until mass constancy to determine mass loss.

To simulate total tract digestibility of CU, mass loss of urea was determined after a 2‐step in vitro process following rumen protection evaluation, aiming to mimic in vivo abomasal and small intestinal incubation for a duration of 2 h and 24 h, respectively. For the abomasal incubation simulation, the residue from the first step (the rumen stability test) was quantitatively transferred to a 1000‐ml bulkhead bottle containing 250 ml of a hydrochloric acid solution (pH = 2.0) containing pepsin. The mixture was preheated to 39°C and incubated for 2 h at 100 rpm with an amplitude of 25 mm (horizontal circular movement). After incubation, the contents were filtered and the residue was washed with 20 ml of ice‐cold water. For simulation of small intestinal incubation, this residue was then added to 250 ml of prepared pancreatic solution (contained 120 mg pancreatin, ≥8 USP lipase units/mg) and incubated for 24 h at 39°C and 100 rpm with an amplitude of 25 mm (horizontal circular movement). After incubation, the contents of the bottle were filtered, washed with cold water, and dried at 39°C until mass constancy to determine mass loss. The CU rumen protection and digestibility were calculated as 1000 – urea mass loss, where urea mass loss (g/kg) was expressed as a proportion of initial amount of urea in the starting sample of CU.

**Table 2:** Product characteristics of urea (U) and coated urea (CU) and in vitro evaluation of CU used in the experiment (g/kg).

	Product	
Characteristic	U	CU ( ± standard deviation)
Urea content	≥980^a^	890 ± 3.4
Fat content	ND^b^	110 ± 3.4
Ruminal release^c^	ND	272 ± 4.0
Digestibility^c^	ND	929 ± 13.2

^a^ According to manufacturer's product specifications (Yara, Brunsbüttel, Germany).

^b^ ND, not determined.

^c^ According to in vitro evaluation. Mass loss of CU for determining ruminal release was obtained after a 6‐h in vitro simulated rumen incubation. Mass loss of CU for digestibility was determined after a 2‐step in vitro process following the simulated rumen incubation, aimed to mimic in vivo abomasal and small intestinal incubation for a duration of 2 h and 24 h, respectively. See text for details. Mass loss (g/kg) was expressed as a proportion of initial amount of urea in CU.

### 2.3 Diet preparation

All non‐roughage components of the diet (Table 1) were mixed as a compound feed in 1‐ton batches using a Nauta Mixer (ABZ Diervoeding, Leusden, the Netherlands). Subsequent TMR mixing (compound feed plus roughages) was performed immediately before daily feeding time at the dairy research farm. Before the animal experiment, compound feed homogeneity tests, based on the coefficient of variation of N, were conducted at the feed mill to establish mixing times required for suitable homogeneity. Based on these tests, a mixing time of 180 s after addition of the final ingredient (i.e., soybean meal, urea, or CU) was found to result in adequate mixability (CV < 5%), and all experimental compound feed batches were prepared according to this standard. Similarly, homogeneity tests were performed on‐farm to establish mixing times required for sufficient homogeneity of the final TMR (CV < 5%), based on the coefficient of variation of N. Each treatment TMR was prepared on‐farm using a horizontal TMR mixer, and diets were mixed for 150 s after final ingredient addition (i.e., compound feed) based on the results of the homogeneity tests. The TMR was delivered to the RIC bins immediately after mixing.

### 2.4 Measurements and chemical analysis

Composite samples of ingredients used to determine TMR composition were collected on days 17 and 19 of each period (covariate, PR, and FR). Samples were frozen immediately at ‐18°C until analysis. Cows were milked twice daily, at 0530 h and 1630 h, and milk yield was recorded electronically. Milk samples were obtained via automatic samplers in the milking parlour collecting a fixed volume of milk per kilogram produced during the morning and evening milking of days 19 and 21 of each period. Samples were collected per cow and per milking into tubes containing sodium azide and bronopol as preservatives, stored at 4°C, and analysed within 3 d. Body weight (BW) and body condition score (BCS) were automatically recorded daily after evening milking when cows exited the milking parlour. The BCS recording was done with a body condition scoring camera system (DeLaval, Tumba, Sweden). A set of 10 blocks of multiparous cows were selected for blood sampling for determination of plasma urea concentrations. Blood sampling was conducted on a first subset of five blocks (15 cows) on day 19 and the other subset of blocks on d 21 during the PR and FR periods. Sampling occurred at 08:00, 10:00, 12:00 and 14:00 h, thus representing 1 h before feeding and 1, 3 and 5 h after feeding, respectively. Samples were collected from the coccygeal vessels into sodium heparin vacutainers and immediately placed on ice, after which vacutainer tubes were centrifuged at 1500 × *g* for 10 min at room temperature. Plasma samples were aliquoted into vials and immediately frozen at −20°C until analysis.

Preparation and analyses of feed samples for moisture, ash, N, NH_3_, NDF, ADF, ADL, starch, total sugar, and crude fat were conducted according to the methods described by Rauch et al. (2021). Plasma urea concentration was analysed using the urea liquicolor test (HUMAN, Wiesbaden, Germany), based on measuring light absorbance at 578 nm after a modified Berthelot reaction. Milk samples were analysed by mid‐infrared spectroscopy according to Rauch et al. (2021).

### 2.5 Calculations and statistical analysis

Milk yield and milk composition data were averaged for the final 7 days of each period. Fat‐ and protein‐corrected milk (FPCM, kg/d) was calculated as [0.337 + 0.116 × milk fat (%) + 0.06 × milk protein (%)] × milk yield (kg/d) (CVB Centraal Veevoederbureau, 2018). Feed efficiency was calculated as kg FPCM/kg DMI. Milk N efficiency (%) was calculated as (CP in milk/6.38)/(CP intake/6.25) × 100, where CP in milk and CP intake refer to total composite CP in milk and total composite CP in feed during the last 7 d of each period, respectively. Human‐edible protein (HEP) efficiency was calculated as [(total HEP output in milk)/(HEP intake) × 100], according to HEP contents of ingredients as reported by Wilkinson (2011). The HEP portion of milk protein was calculated based on milk true protein content (milk CP × 94.5%; based on an estimated NPN content of milk of 5.5% (DePeters & Ferguson, 1992). Cumulative DMI for each hour post‐feeding are based on the final 7 days of each period according to the following formula:

$${\left[\left(\text{cumulative}\;{\text{DMI}}_{t}/\text{total}\;\text{DMI}\right)\times 100\right]}_{T}–{\left[\left(\text{cumulative}\;{\text{DMI}}_{t}/\text{total}\;\text{DMI}\right)\times 100\right]}_{SBM}$$

where t denotes hour post‐feeding and *T* denotes the treatments U and CU, respectively. This allowed relative changes in DMI responses of each urea source to be reported relative to that of SBM.

**Figure 2:**
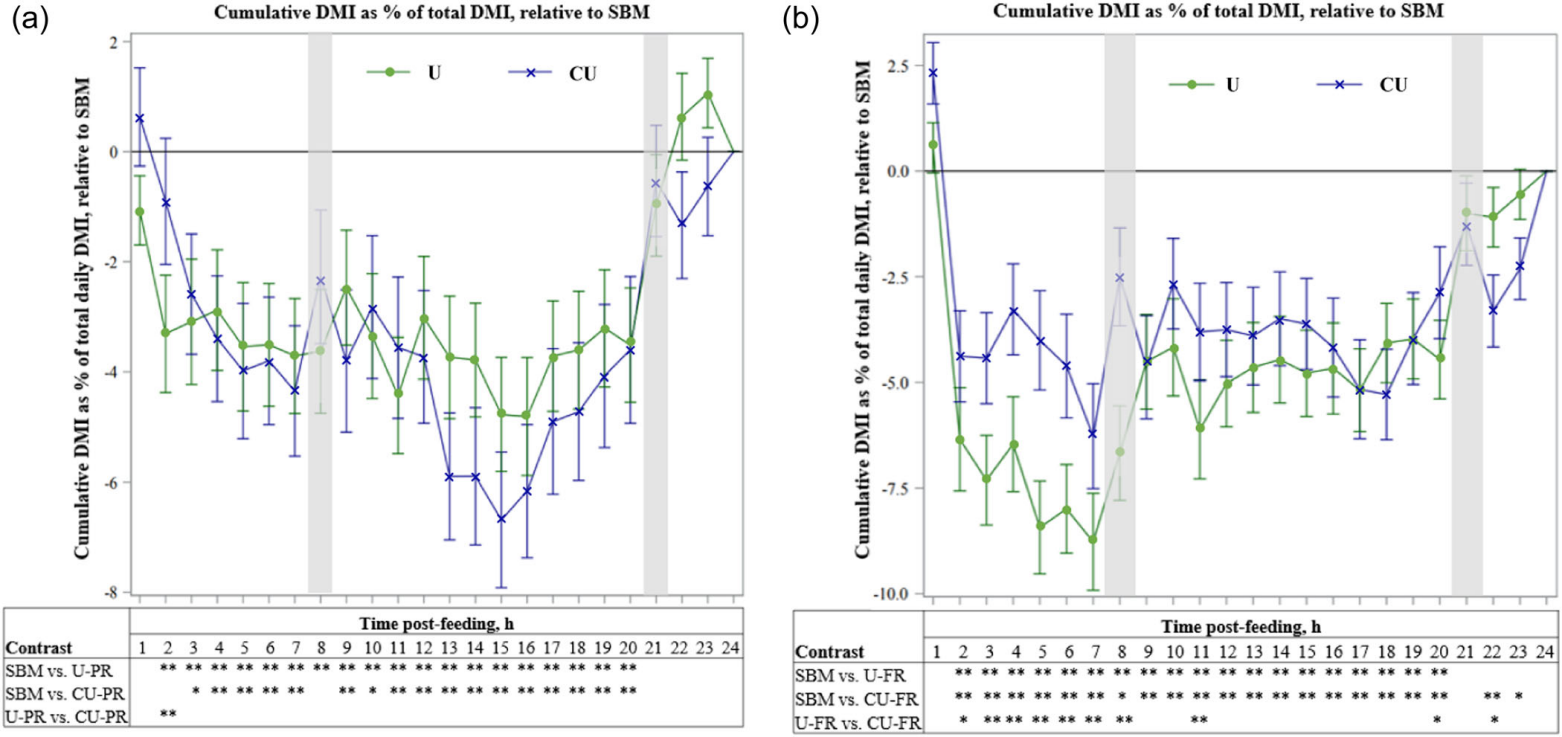
Relative differences in dry matter intake (DMI) of lactating dairy cows by hour after feeding (0900 h) between a TMR based on soybean meal (SBM) or TMR replacing of a portion of SBM with urea (U) or coated urea (CU) at (a) 12 g urea equivalent/kg dry matter (partial replacement; PR) or (b) 19 g urea equivalent/kg dry matter (full replacement; FR). Values for each hour are calculated as: [(cumulative DMI_t_/total DMI)×100]_*T*_ – [(cumulative DMI_t_/total DMI)×100]_*SBM*_ where t denotes hour post‐feeding and *T* denotes the treatments U and CU, respectively. Data from the last 7 days of the partial replacement period was used. *0.05 < *p* < 0.10; tendency for difference between treatments. **p ≤ 0.05; significant difference between treatments. Error bars indicate SEM and shaded areas indicate milking times.

All statistical analyses were conducted with PROC MIXED in SAS (SAS 9.4M6, SAS® Studio, SAS Institute Inc., Cary, NC, USA), with cow as the experimental unit. Cow, treatment, and block were used as class variables. The statistical analysis was performed for the partial replacement and full replacement periods separately. Data on intake, production, and plasma urea concentrations were averaged per cow and period. The model included the fixed effects of treatment and the associated covariate value (for all data except plasma urea concentrations), and block was included as random effect. For analysis of differences by hour (i.e., plasma urea and feed intake pattern), the SLICE statement of SAS was used to correct for multiple comparisons of the 3 treatments within an hour. For all data, if a value had a studentized residual of less than −3 or more than 3, it was classified as outlier and removed before statistical analysis. For all statistical analyses, differences were considered significant at *p* ≤ 0.05 and tendencies at 0.05 < *p* ≤ 0.10.

## 3 RESULTS

### 3.1 In vitro product evaluation and product characteristics

The CU product contained 890 g urea/kg and 110 g fat coating/kg, whereas U contained no fat and ≥980 g urea/kg (Table 2). Ruminal release and total tract digestibility of CU was estimated to be 272 and 929 g/kg, respectively, based on an in vitro evaluation of the CU product before the in vivo experiment. Based on these results, the CU product was considered sufficiently rumen‐stable and digestible to test the hypothesis in the in vivo experiment.

### 3.2 Intake, milk production, body weight and nutrient efficiency

Dry matter intake, CP intake, and yields of milk, milk fat, milk CP, and FPCM decreased (*p* ≤ 0.03; Table 3) and milk concentration of lactose and urea increased (*p* ≤ 0.02) with U‐PR compared with SBM. Body weight was lower (*p* = 0.01) and HEP efficiency was higher (*p* < 0.01) in response to U‐PR compared with SBM. Dry matter intake, CP intake, yields of milk and milk CP, and milk protein concentration decreased (*p* ≤ 0.03) and FPCM tended (*p* = 0.08) to decrease with CU‐PR compared with SBM. Milk lactose content, urea content, and HEP efficiency increased (*p* ≤ 0.02), and BW decreased (*p* < 0.01) with CU‐PR compared with SBM. No differences in intake, milk production, BW, or nutrient efficiencies were observed between U‐PR and CU‐PR (*p* ≥ 0.13).

**Table 3:** Performance and plasma urea concentrations of lactating dairy cows (*n* = 18/treatment) receiving TMR based on soybean meal (SBM) or isonitrogenous partial replacement (PR) or full replacement (FR) of SBM with urea (U) or coated urea (CU).

	Treatment^a^							*P*‐value					
	Partial replacement			Full replacement				Partial replacement			Full replacement		
Parameter	SBM	U‐PR	CU‐PR	SBM	U‐FR	CU‐FR	SEM	SBM versus U‐PR	SBM versus CU‐PR	U‐PR versus CU‐PR	SBM versus U‐FR	SBM versus CU‐FR	U‐FR versus CU‐FR
DMI (kg/d)	25.7	23.0	23.8	25.8	22.5	22.7	0.52	<0.01	0.02	0.54	0.01	<0.01	0.97
CP intake (kg/d)	4.12	3.71	3.76	4.15	3.67	3.54	0.083	<0.01	<0.01	0.90	<0.01	<0.01	0.52
Yield (kg/d)													
Milk	29.0	26.7	26.8	29.7	23.6	23.3	0.45	<0.01	<0.01	0.99	<0.01	<0.01	0.91
Fat	1.33	1.21	1.30	1.40	1.20	1.20	0.04	0.03	0.78	0.13	<0.01	<0.01	1.00
CP	1.13	1.00	1.00	1.16	0.87	0.86	0.02	<0.01	<0.01	1.00	<0.01	<0.01	0.98
Lactose	1.28	1.21	1.21	1.33	1.06	1.05	0.03	0.11	0.15	0.99	<0.01	<0.01	0.92
Fat‐ and protein‐corrected milk^b^	31.8	29.0	30.1	33.1	27.4	26.9	0.58	<0.01	0.08	0.35	<0.01	<0.01	0.86
Milk composition													
Fat (%)	4.65	4.64	4.82	4.63	5.23	5.24	0.11	1.00	0.28	0.26	<0.01	<0.01	1.00
CP (%)	3.86	3.76	3.74	3.91	3.74	3.70	0.04	0.10	0.03	0.89	0.01	<0.01	0.71
Lactose (%)	4.41	4.52	4.52	4.45	4.51	4.51	0.03	0.02	0.02	1.00	0.17	0.14	1.00
Urea (mg/dL)	15.8	19.5	19.0	14.2	22.4	23.6	0.76	<0.01	<0.01	0.73	<0.01	<0.01	0.41
BCS	3.05	3.04	3.05	3.12	3.09	3.10	0.02	0.84	0.99	0.92	0.47	0.65	0.96
BW (kg)	690	680	678	695	671	676	4.00	0.01	<0.01	0.78	<0.01	0.01	0.68
Feed efficiency (kg FPCM/kg DMI)	1.26	1.27	1.26	1.30	1.20	1.18	0.03	0.91	1.00	0.92	0.09	0.03	0.80
Milk N efficiency (%)^c^	27.2	26.6	26.6	27.4	23.2	24.1	0.68	0.66	0.15	0.52	<0.01	<0.01	0.66
Human edible protein efficiency (%)^d^	63	125	122	62	207	204	3.83	<0.01	<0.01	0.80	<0.01	<0.01	0.84
Plasma urea (m*M*)	4.65	5.26	4.90	4.82	6.16	6.29	0.163	<0.01	0.25	0.07	<0.01	<0.01	0.62

^a^ Partial replacement of SBM with U (U‐PR) or CU (CU‐PR) was at 12 g/kg urea equivalents and full replacement of SBM with U (U‐FR) or CU (CU‐FR) was at 19 g/kg urea equivalents (all DM basis).

^b^ FPCM (fat‐ and protein‐corrected milk) calculated using the formula: [0.337 + 0.116 × milk fat (%) + 0.06 × milk protein (%)] × milk yield (kg/d) (CVB Centraal Veevoederbureau, 2018).

^c^ Milk nitrogen efficiency (%) = [(milk protein output/6.38)/(dietary CP intake/6.25)] × 100.

^d^ Human edible protein efficiency calculated as [(total human edible protein output in milk)/(human edible protein intake) × 100], according to human edible protein contents of ingredients as reported by Wilkinson (2011).

Dry matter intake, CP intake, yields of milk, milk fat, milk CP, milk lactose, FPCM, and milk CP concentration decreased with U‐FR compared with SBM (*p* ≤ 0.01). Milk concentration of fat and urea increased (*p* < 0.01), BW and milk N efficiency decreased (*p* < 0.01), and feed efficiency tended (*p* = 0.09) to decrease with U‐FR compared with SBM. Human‐edible protein efficiency increased (*p* < 0.01) with U‐FR compared with SBM. Dry matter intake, CP intake, yields of milk, milk fat, milk CP, milk lactose, FPCM, and milk CP concentration decreased (*p* < 0.01) and the concentration of milk fat and urea increased (*p* < 0.01) with CU‐FR compared with SBM. Body weight, feed efficiency, and milk N efficiency decreased (*p* ≤ 0.03) with CU‐FR compared with SBM. Human‐edible protein efficiency increased (*p* < 0.01) with CU‐FR compared with SBM. No differences in intake, milk production, BW, or nutrient efficiencies were observed between U‐FR and CU‐FR (*p* ≥ 0.41).

### 3.3 Cumulative DMI

Cumulative DMI of U‐PR was lower (*p* < 0.05) compared with SBM from hour 2 to 20 post‐feeding (Figure 2a). Cumulative DMI tended (*p* < 0.10) to be lower during hour 3 and 10 post‐feeding and was lower (*p* < 0.05) during hour 4 to 7, hour 9, and hour 11 to 20 post‐feeding for CU‐PR compared with SBM. The greatest difference in cumulative DMI with U and CU relative to SBM was at approximately hour 15 post‐feeding. Cumulative DMI was higher within the first 2 h after feeding with CU‐PR compared with U‐PR (*p* < 0.05).

Cumulative DMI was lower (*p* < 0.05) during hour 2 to 20 post‐feeding for U‐FR compared with SBM (Figure 2b). Cumulative DMI was lower (*p* < 0.05) for CU‐FR compared with SBM during hour 2 to 7, 9 to 20, and 22, and tended (*p* < 0.10) to be lower for hour 8 and 23 post‐feeding. Compared with U‐FR, CU‐FR had greater cumulative DMI (*p* < 0.05) during hour 3 to 8 and hour 11 and tended (*p* < 0.10) to be greater during hour 2 and 20 post‐feeding. The lowest cumulative DMI with U and CU relative to SBM was at approximately hour 7 post‐feeding. Cumulative DMI during hour 22 post‐feeding tended (*p* < 0.10) to be lower for CU‐FR compared with U‐FR.

### 3.4 Plasma urea concentration

**Figure 3:**
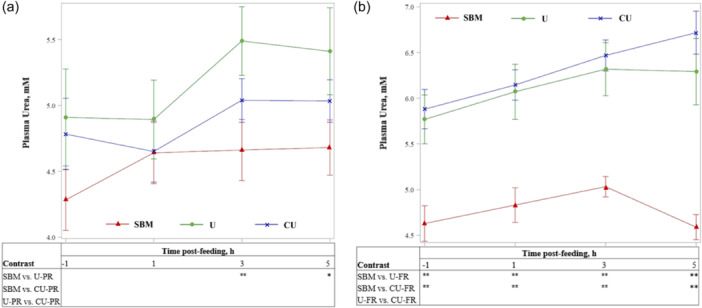
Plasma urea concentrations in response to a TMR based on soybean meal (SBM) or TMR replacing of a portion of SBM with urea (U) or coated urea (CU) at (a) 12 g urea equivalent/kg dry matter (partial replacement; PR) or (b) 19 g urea equivalent/kg dry matter (full replacement; FR). Blood samples were taken at ‐1, 1, 3 and 5 h relative to feeding (0900 h). *p* ≤ 0.05 for treatment and hour; no significant treatment × hour interaction (*p* > 0.14). For hourly contrasts: *0.05 < *p* < 0.10, tendency for difference between treatments; **p ≤ 0.05, significant difference between treatments. Error bars indicate SEM.

Average plasma urea concentration increased with U‐PR compared with SBM (*p* < 0.01; Table 3) and tended to be higher with U‐PR compared with CU‐PR (*p* = 0.07). Over time, plasma urea concentration was greater (*p* < 0.05) with U‐PR compared with SBM 3 h post‐feeding and tended (*p* < 0.10) to be greater 5 h post‐feeding (Figure 3a). Plasma urea concentration did not differ with CU‐PR compared with SBM at any time point. Average plasma urea concentration increased with U‐FR and CU‐FR over SBM (*p* < 0.01; Table 3), and did not differ between U‐FR and CU‐FR. Over time, plasma urea concentrations were higher (*p* ≤ 0.05) with U‐FR and CU‐FR compared with SBM during ‐1, 1, 3 and 5 h relative to feeding (Figure 3b). Plasma urea concentrations did not differ between U and CU at any time point or replacement level.

## 4 DISCUSSION

### 4.1 In vitro product evaluation

Based on the results of the in vitro evaluation, the CU product was expected to have a high degree of in vivo rumen protection and total‐tract digestion. The in vitro ruminal release of CU of 27% after 6 h observed in the current study is lower than that from other CU products after only 1 h in the studies of Ravi Kanth Reddy et al. (2019; 86%) and Galo et al. (2003; 83%), and lower than the disappearance after a 0.5‐h in sacco incubation reported by Highstreet et al. (2010; 72%).

### 4.2 Urea and coated urea versus soybean meal

Average DMI was reduced 11 and 7% in response to U‐PR and CU‐PR, respectively, compared with SBM. Decreased DMI in response to urea can be expected with high inclusion levels (e.g., >10 g/kg DM; Nichols et al., 2023; Polan et al., 1976; Santos et al., 1998). As expected, DMI was reduced by a larger margin during FR compared with PR, where average DMI was reduced 13 and 12% in response to U‐FR and CU‐FR, respectively, compared with SBM. Hypophagic effects of urea supplementation have been attributed to poor palatability of urea (i.e., taste; Huber & Cook, 1972) or consequences of increased ruminal ammonia concentrations (e.g., ammonia trapping in epithelial cells, reduced rumen motility; Conrad et al., 1977; Davidovich et al., 1977). However, others (Nichols et al., 2023; Wilson et al., 1975) have reported that post‐ruminal urea supplementation at levels approaching or exceeding 2% of dietary DM also decreased DMI of dairy cattle, suggesting that factors other than palatability play a role in reducing DMI when urea‐containing diets are fed. These findings agree with our observations where both ruminally and post‐ruminally available urea supplemented at 1.2 and 1.9% of dietary DM reduced DMI compared with iso‐nitrogenous SBM. Given that urea, whether degraded in the rumen or post‐rumen, will produce more ammonia compared with SBM (Teller & Godeau, 1984), the amount of ammonia absorbed into portal circulation and subsequently reaching the liver for detoxification to urea would have increased when U or CU replaced SBM. Bacterial urease activity has been identified in the digesta and mucosa of the post‐ruminal gastrointestinal tract of sheep (abomasum through colon; Marini et al., 2004; Michnová et al., 1979; Whitelaw et al., 1991), thus we assume urea from the CU product that arrived post‐ruminally was hydrolysed and absorbed as ammonia. Increasing hepatic ammonia load has been shown to increase oxidative catabolism of amino acids and propionate (Brosnan & Brosnan, 2009; Demigné et al., 1991; Milano & Lobley, 2001), which may be associated with elevated hepatic ATP concentrations translating a neural satiety signal to the centre of the brain influencing feeding behaviour (Allen et al., 2009). Taken together, decreased DMI with U and CU compared with SBM at both replacement levels could have been related to ruminal signals, post‐absorptive signals, or a combination of these factors, although the magnitude of contribution of these mechanisms between urea sources and replacement levels cannot be delineated based on the current study.

There is evidence that microbial function and subsequently fibre fermentation is compromised when the contribution of urea to rumen‐degradable protein increases (Broderick & Reynal, 2009). This is consistent with suggestions that certain rumen micro‐organisms need or make use of amino acids and peptides (Bach et al., 2005), and that the efficiency of microbial protein synthesis is lower when microbes use exclusively ammonia compared with amino acids and peptides (Dijkstra et al., 1998). Although quantifying precise contributions of amino acids and peptides to microbial metabolism is difficult due to cross‐feeding between microbial species (Nolan & Dobos, 2005), it is possible that the reduction in DMI in response to U and CU compared to SBM observed in the current study was related to a concomitant reduction in ruminal availability of amino acids or peptides when replacing SBM with U and CU, and subsequent negative effects on fibre digestibility and rumen fill. Further, the NDF content of the TMR for U and CU was numerically higher (7% and 13% for PR and FR, respectively, on average across U and CU) than the TMR for SBM. Combined with potentially reduced capacity for microbial fermentation, the higher NDF content may have negatively influenced DMI (Daniel et al., 2016).

Compared with SBM, milk and milk protein yield decreased in response to U and CU at both levels of SBM replacement. Other studies similarly report reductions in yield of milk and milk protein in response to relatively high urea supply (Brito & Broderick, 2007; Broderick & Reynal, 2009; Nichols et al., 2023). Reductions in milk production in the current study and those of others are driven largely by hypophagic effects of high dietary urea inclusion. However, it is intriguing that PR reduced DMI by 9% and milk yield by 8% with U and CU, on average, whereas FR reduced DMI by 12% but milk yield was reduced by 21% with U and CU, on average. Similarly, milk protein yield was reduced by 12% with PR and by 25% with FR, on average across U and CU. In addition to the effect of FR on DMI, it seems the higher level of urea inclusion also impacted nutrient digestibility or utilisation such that milk and milk protein yield decreased to a relatively greater extent, regardless of urea source. In contrast, the reduction in milk fat yield was more in line with the reduction in DMI across the urea sources (6% for PR and 14% for FR), suggesting that milk fat synthesis was less affected by a possible shift of mammary nutrient availability induced by dietary urea compared with milk protein synthesis. Body reserves could have been partially contributing to milk protein and fat production, as BW was lower with U and CU compared with SBM at both replacement levels. Similarly, Broderick and Reynal (2009) reported a linear decrease in BW gain as urea replaced a greater portion of rumen‐degradable protein in diets of dairy cattle. In line with the relatively larger decrease in milk and milk protein production at FR compared with PR for U and CU, feed and milk N efficiency did not differ between SBM, U, and CU during PR, but were lower with U and CU compared with SBM during FR (only tendency for U‐FR compared with SBM). Further, if full replacement of SBM with U or CU increased rumen ammonia levels to a greater extent relative to microbial requirements, we would expect a reduced milk N efficiency given the negative relationship between rumen ammonia concentration and the efficiency of N utilisation of rumen microbes (Bach et al., 2005). Although CU was designed to release most of the urea post‐ruminally, a portion was rumen‐available, and the higher supply of the CU product at FR likely increased rumen ammonia concentrations to a greater extent than at PR.

### 4.3 Feed intake pattern with urea versus coated urea

In contrast to our hypothesis, we observed no difference in total daily DMI between U‐PR and CU‐PR, or between U‐FR and CU‐FR. This is consistent with some studies in which urea was replaced by a CU source without effects on DMI (Highstreet et al., 2010; Sinclair et al., 2012), but not with observations from Xin et al. (2010) who reported increased DMI for a CU source compared to urea. Generally, commercial CU sources aim to still supply urea to the rumen but in a slow‐release form, contrasting our aim to shift the majority of the dietary urea from the rumen to the post‐ruminal gut with the CU product. Variability in responses may be partly explained by different basal diet characteristics between studies (e.g., dietary CP level and degradability of energy and protein sources), differences in coating characteristics between CU products, level of dietary urea inclusion or rate of urea infusion, or combinations of these factors.

Others have reported differences in feed intake pattern when dietary urea inclusion approaches or exceeds 1% of dietary DM (Kertz et al., 1982; Sinclair et al., 2000). With that in mind, we aimed to assess DMI over 24 h post‐feeding to determine if the reductions in DMI with U and CU compared with SBM arose from different intake patterns between the urea sources. Despite the similar total daily DMI between U and CU at both replacement levels, the cumulative DMI relative to SBM over the 24‐h post‐feeding period differed between the urea sources. During PR, we observed a greater cumulative DMI for CU versus U during 1 h per 24‐h period (2 h post‐feeding). During FR, cumulative DMI was greater (significant or tendency) with CU compared with U for 9 h per 24‐h period, most consistently during the 7 h post‐feeding, and tended to be smaller 3 h before the next feeding period. The overall pattern of DMI during FR (Figure 2b) suggests DMI relative to SBM on U and CU decreased up to 7 h post‐feeding and increased until the next fresh feed allocation, but that this initial decrease in relative DMI rate was more pronounced with U than CU. Differences in feed intake pattern between U and CU at PR and FR may have resulted from shifts in the dynamics of ammonia absorption due to differences in rate and site of urea release between U and CU subsequently impacting metabolic feedback regulating feed intake (as discussed above).

Plasma urea concentrations were higher with U‐PR compared to SBM at 3 h and 5 h (tendency only) post‐feeding but did not differ between CU‐PR and SBM, whereas plasma urea concentration increased with U‐FR and CU‐FR over SBM on average and at all individual time points. This difference in concentration relative to SBM with U and CU at different inclusion levels suggests a difference in rate or site of release between urea sources as their inclusion increases. Plasma urea concentration reflects the amount of urea in circulation at a given moment but does not describe the relative fluxes of urea and ammonia that may be occurring across the gastrointestinal tract and post‐absorptive tissues.

### 4.4 Human‐edible protein efficiency

Recently, there has been increased focus on the capacity of ruminants to convert human‐inedible ingredients into high quality human‐edible food such as milk protein (Broderick, 2018; Dijkstra et al., 2013; Wilkinson, 2011). Increased focus on this characteristic strengthens the position of ruminants in global food production systems by highlighting their ability to upcycle ingredients that humans and monogastric animals cannot use for anabolic purposes (e.g., urea). When comparing U and CU with SBM in the current study, HEP efficiency approximately doubled with PR and tripled with FR. The values exceeding 100% for U and CU at both PR and FR highlight the ability of ruminants be net contributors to HEP supply.

## 5 CONCLUSION

We hypothesised that hypophagic effects of high dietary urea inclusion would be mitigated by CU, a product designed to partially shift urea supply from the rumen to the post‐ruminal gut. We observed hypophagic responses when SBM was replaced by U or CU at both partial (12 g urea equivalent/kg DM) or full (19 g urea equivalent/kg DM) replacement. Negative effects of dietary inclusion of urea on DMI were not different between urea sources. However, particularly in the initial hours post‐feeding, cumulative DMI with U and CU relative to SBM decreased to a greater extent with U compared with CU in the full replacement period. This difference in feed intake pattern suggests differences in rate or location of N availability between urea sources. Milk yield, milk protein yield, and BW decreased with U and CU compared with SBM at both replacement levels. Plasma urea concentration was higher with U compared with SBM at partial replacement and was higher with U and CU compared with SBM at full replacement. Milk N efficiency did not differ between SBM, U, and CU at partial replacement, but decreased with U and CU compared with SBM at full replacement. Milk production, milk composition, BW, and feed and milk N efficiency were not different between urea sources during partial or full replacement. Replacing SBM partially or fully with urea sources increased HEP efficiency.

## 6 CONFLICT OF INTEREST STATEMENT

This study was funded by Trouw Nutrition (Amersfoort, the Netherlands), a company with commercial interests in dairy cattle nutrition. R. Rauch, K. Nichols, I. P. C. de Carvalho, J. B. Daniel and J. Martín‐Tereso were employed by Trouw Nutrition. Trouw Nutrition R&D is committed to high standards of research integrity and adheres to the principles of the European Code of Conduct for Research Integrity (Drenth, 2012).

## Data Availability

Data from this work may be made available upon request to the corresponding author.
